# Relationship Between Teachers’ Teaching Modes and Students’ Temperament and Learning Motivation in Confucian Culture During the COVID-19 Pandemic

**DOI:** 10.3389/fpsyg.2022.865445

**Published:** 2022-05-26

**Authors:** Chuan-Yu Mo, Jiyang Jin, Peiqi Jin

**Affiliations:** ^1^School of Education and Music, Sanming University, Sanming, China; ^2^Business School, Beijing Normal University, Beijing, China; ^3^College of Foreign Languages, Zhejiang University of Technology, Hangzhou, China

**Keywords:** temperament, learning motivation, teaching model, Confucian heritage culture settings, COVID-19

## Abstract

Because of the coronavirus disease 2019 (COVID-19) pandemic, the traditional didactic teaching method that is practiced in Confucian culture, an Eastern cultural model, is being challenged by multiple alternative teaching modes. In Western cultures, the teaching behavior of teachers is dependent on their ability to influence the temperament of students; in contrast, teachers in Eastern cultures are influenced by changes in external environment (i.e., social policy). This phenomenon can mainly be explained by the tendency of students in Eastern cultures to adopt a passive learning style because of Confucianism. On the basis of Confucian culture and literature review, we conducted a Chinese-language questionnaire survey of temperament scales and learning motivation, and 724 effective questionnaires were collected and used to explore the relationship between students’ temperament and learning motivation under three teaching modes, namely, online teaching, traditional offline classroom teaching, and online–offline mixed teaching. Conclusions drawn were as follows. (1) In a Chinese Confucian cultural system, the passive learning style of students and its relationship with the surrounding collective culture creates the temperament characteristics of sanguinity and phlegmatism. (2) Influenced by the traditional Confucian values of benevolence and honesty, students with a melancholic temperament do not focus on their learning motivations. Furthermore, changes in external information, to which such students are sensitive, cause them to give up opportunities easily. (3) Similarly, students with a choleric temperament are sensitive and prone to fluctuating emotions, and they tend to be affected by changes in their external environment. (4) Although students have a strong learning motivation under the traditional offline teaching mode, a paradox in individual cognition exists because of differences between Chinese and Western cultures. Therefore, students generally prefer the online–offline mixed teaching mode to the traditional single teaching mode. This study explored factors that influence students’ learning motivation, namely, individual temperament and educational environment (e.g., teaching modes), and provides a reference for the future development of post epidemic education.

## Introduction

After the coronavirus disease 2019 (COVID-19) pandemic started in 2020, the topic of high-quality teaching modes in higher education attracted widespread attention, and changes occurred to the traditional offline teaching mode. In response to the outbreak of the epidemic, the Ministry of Education of China implemented a new teaching mode in schools at all levels (Cheng, 2020; Zhang et al., 2020). Specifically, all courses were taught online in a short time frame. After the epidemic eased in China in 2021, the Chinese teaching mode became an online–offline hybrid model, which not only promoted the transformation of higher education but also met the various needs of school or external environments (Toe, 2008). In the face of these rapid changes, students were forced to invest their time and energy in familiarizing themselves with innovative teaching modes; consequently, learning attitudes, and behavioral intentions of students with respect to the long-term implementation of various teaching modes (e.g., online teaching) became a topic of concern (Lin et al., 2021).

Studies have reported that students who are deeply influenced by traditional Confucian culture do not like the online teaching mode and prefer the traditional teaching mode (i.e., a single offline face-to-face mode; Chan, 2019). However, this type of traditional single teaching mode is not favored by some Western scholars who have argued that it is not conducive to learning (Fan et al., 2004). However, students who are influenced by Confucianism have performed well in memory, repetition, and problem-solving tasks, such as mathematics-related tasks that require memory and long-term logical thinking skills. Thus, scholars are increasingly exploring this Confucianism-oriented teaching mode, which is influenced by collectivism, high parental expectations, attribution of effort (Wong, 2008), and obedience to authority (O’Dwyer, 2016). In this mode, collectivism is regarded as a key reason for the high learning motivation of Chinese students (Dasari, 2009). These students are obedient within the framework of a unified teaching arrangement, and they refrain from sharing their opinions to avoid conflicts violating group norms (Turner, 2011). As passive recipients, these students have a learning style that is different from the autonomous learning style of students in Western countries.

Learning strategies that students adopt are related to their teachers’ teaching methods (Morrison, 2006). Moreover, these learning strategies are associated with learning motivation, which can enhance students’ thinking ability in the context of their learning styles (Fontes, 2016). Because teachers in Eastern countries tend to assume that students can only absorb information through rote learning, they also tend to implement new teaching methods on the basis of this assumption (Dasari, 2009; Sakurai et al., 2014). Consequently, classroom activities indirectly focus on the value of learning results instead of learning enjoyment (Choi and Cho, 2020). Therefore, students have different views of learning objectives. However, Elliot and Thrash (2002) asserted that these perceptions are due to differences in temperament among students who are innately sensitive to extrinsic rewards and punishments. A student’s temperament (including emotion and orientation) is closely related to his relationship with teachers (Buonomo et al., 2017). Thus, teachers in Western countries focus on whether their teaching behavior influences the temperaments of their students during implementation of a teaching mode. However, because teachers and students in Eastern countries are influenced by Confucianism, their teaching mode is mainly dependent on changes in their external environment (i.e., social policy). In contrast, student-related factors are not considered during the initial implementation of teaching modes (Bliesener and Adelmann, 2000). Whether students can normally modify their own learning mode (i.e., learning motivation) is determined by interactions between their personal characteristics (self-factors) and situations they are exposed to (external environment; Rheinberg, 2000).

In summary, during the COVID-19 epidemic, China’s teaching mode was mainly based on policies and the external environment, and it did not prioritize the personal characteristics of students, resulting in a diversified, changeable, and mixed teaching mode. Whether students can adapt to these changes should be a key consideration for teachers. However, in Confucian culture, which focuses on instructions of the incumbent government, specific social and economic values are consequently emphasized; Confucian culture also embodies the spirit of humanitarianism (Chin et al., 2021a), meaning that it focuses on needs and motives of individuals. Therefore, this study examined the relationship between students’ learning motivation and their temperament under various teaching modes from the perspective of Chinese Confucian culture and conducted a literature review of Confucian culture–related research to explore the following three questions:

RQ1: What are the differences in learning motivation among students with different temperaments?
RQ2: What are the differences in the learning motivation of students under different teaching modes?
RQ3: What are the motivational behaviors of students under different combinations of temperament and teaching mode?

## Literature Review

### Triadic Reciprocal Determinism

Scholars have argued that human behavior is mainly determined by some factors. Therefore, in a single causal model, a person’s behavior is often attributed to the environment or their internal character. However, Bandura (1989a) proposed a two-way relationship of influence between human behavior and the external environment. From the perspective of social cognition, human nature can shape limitless possibilities through direct or limited alternative experiences. In this causality model, human spontaneous activity is regarded as the core element and decisive determinant of human motivation (Bandura, 1989b), which cannot be determined solely by intrinsic or extrinsic factors. The core idea of social cognitive theory is triadic reciprocal determinism (TRD), which asserts that the acquisition, maintenance, and change of individual behavior is the result of the joint action of the individual, their behavior, and the environment. Behavior, cognition, and environment are interrelated and influenced by each other. It is the interaction among these three factors that is crucial rather than any combination of two factors (Bandura, 1986).

Few studies have applied TRD to explore learning behavior. This study mainly collected studies that explored learning environments in the context of Chinese students during the COVID-19 pandemic (Table 1A).

**TABLE 1A:** Triadic reciprocal determinism (TRD)-related studies.

Authors	Research contexts	Content	Constructs
Greener et al. (2007)	Virtual environment	This paper explores how higher education teachers can stimulate and increase students’ interaction in academic debate in a virtual learning environment	Thinking + emotional + behavior + environment
Atas et al. (2017)	Teaching environment	By observing the interactive process of question-answer between teachers and students in classroom teaching, the author obtains students’ evaluation of the learning motivation and other effects of this model	Person + environment + behavior
Tirado-Cordero and Hargiss (2017)	Learning environment	This paper discusses how to use PEB theory to lay the foundation for peer counseling learning environment in the instructional design system (ISD)	GSE + environmental cues + behavior + biological determinants
Schiavo et al. (2019)	Adaptive environment	The author uses a mathematical model to describe how the variables of TRD evolve over time, so as to deduce how humans use the regulatory mechanism of self-efficacy to predict behavior results	Person + environment + behavior + CSE
Benight et al. (2018)	Living environment	This paper studies how people respond to great life emergencies, and explains how people’s self-regulation plays a role through PEB	Person (cognitive and affective) + behavioral + environment
Zhang (2021)	Extra learning tasks	This paper discusses that how large or high-risk examinations affect students’ learning in the context of Chinese mainland	Person + environment + behavior + self-regulated + achievement

During the COVID-19 pandemic, college education was conducted in various learning environments (e.g., virtual online environments, offline classroom environments, extracurricular environments, and living environments). Studies have suggested that TRD can be used to explain how students adapt themselves to various environments, because it entails consideration of individuals, behaviors, and environments. In the context of Chinese higher education, the processes of engaging in discussions, asking questions, and providing answers, and conducting teacher–student reflections further reflect the active and passive learning styles of students under Chinese Confucianism. Studies have discussed how the learning enthusiasm (i.e., learning motivation) of students can be stimulated to ensure that they adopt the appropriate learning mode whenever necessary, apply emotional and cognitive adjustments (including perception abilities such as self-efficacy and sense of achievement), and learn to predict and evaluate their own behavioral results. Therefore, for situational learning in a given cultural background, the ability of individuals to adapt is the core element considered, which interacts with the external environment (Confucian culture, teaching mode during the COVID-19 pandemic) and the behavior (learning motivation) of individuals.

### Regulative Theory of Temperament and Temperament Typology

Chess and Thomas (1996) described temperament as a behavior style, a choice of situation, or a behavior of specific stimulus value (Strelau, 1996). It includes components such as individual cognitive activity level, emotion regulation level, and biological rhythm. Because the temperament of an individual influences regulation of their body and guides their future behavior, Strelau (1987) proposed the regulation theory of temperature on the basis of the Pavlovian central nervous system to explain that the nervous and endocrine systems respond to stimuli (including speed). To establish a foundation for explaining the function of temperament, he proposed six temperament characteristics (Strelau, 2008). However, scholars have subsequently adopted four dimensions, namely, emotional reactivity, endurance, briskness, and activity, to study the relationship of temperament with other factors (Jankowski and Zajenkowski, 2009; Bojanowska and Zalewska, 2016). These factors include cultural and individual behavioral results; specifically, scholars have explored how interactions between temperament and experiences pertaining to an external environment (e.g., a specific culture) influence the stable development of an individual’s emotions, motives, and behavioral patterns (Rothbart, 2007). These four dimensions were subsequently combined with the early Hippocratic theory of the four temperaments, namely, the sanguine, melancholic, phlegmatic, and choleric temperaments (Strelau, 2008; Bojanowska and Zalewska, 2016).

For cultural factors, several temperament scales have incorporated cultural specificity into behavior measurement, and they are, thus, often used to evaluate behavior in various cultures (Strelau and Plomin, 2008). When unexpected situations occur, culture and temperament always interact, indicating that temperament leads to cultural problems and culture shapes temperament characteristics (Chen, 2018). In particular, an acquired environment includes the parenting style of parents, communication style of peers, and ecological conditions, all of which are related to the characteristic development of temperament (Eisenberg et al., 2007; Chen and Schmidt, 2015). In addition, individual and group biases in the character of children during their early development are a source of temperament changes (Kagan and Snidman, 1992; Rothbart, 2011). Therefore, studies on individual temperament types in China have mostly examined children and adolescents, reflecting a cross-cultural nature. For example, although no obvious distinction exists between Chinese and American children in terms of their internal and external character, compared with American children, Chinese children prefer to think from a practical perspective and exhibit collectivistic behavior (Oakland and Lub, 2006). However, learning through the Internet has led to some problems for Chinese teenagers. By examining students with the four temperament types, we discovered that the four dimensions of temperament (i.e., perception, shyness, effective control, and anger/frustration) play an intermediary role in determining whether students can use the Internet to learn effectively (Li et al., 2016). As for Chinese college students, they focus on social needs because of the influence of Chinese cultural factors. An individual with a sufficiently fast mental speed can easily adapt to a fast-paced way of life, and individuals who exhibit a high level of perseveration can easily cope with fatigue resulting from activities (Liu et al., 2015).

For individual behavioral outcomes, such as achievement, motivation, and learning goals, individual temperament has been theoretically hypothesized to be related to achievement goals or achievement motivations; however, only few studies have verified this association (Elliot and Thrash, 2002). Chen and Zhang (2011) discussed how temperaments of Chinese high school students affected their individual achievement motivation, and they reported that students adapted in accordance with their temperament and personality characteristics and eventually acquired positive learning values. These values resemble the results of students’ evaluation of self-learning. Whether students are interested in what they have learned is defined as “the individual’s relatively persistent tendency to pay attention to the object”; this definition highlights the role of learning motivation and expectations in a given personality trait (Renninger et al., 1998). In education, different temperaments have different levels of sensitivity to external perception; thus, students have different learning and performance goals. These goals are often related to personal sense of achievement, self-efficacy, and social emotion (Rawlings et al., 2017). Thus, learning motivations of students are influenced in varying degrees (e.g., the behavior mode of self-regulated learning or passive learning), and they reflect the self-regulation model of an individual (Ariyanti and Dahlan, 2019).

With respect to neurophysiological regulation characteristics that are embodied in the four types of temperament, this study examined the following four temperaments from two dimensions, namely, processing capacity and stimulus supply (Jankowski and Zajenkowski, 2009; Bojanowska and Zalewska, 2016):

(1) Choleric temperament: high activity and briskness, low endurance, and high emotional reactivity;

(2) Sanguine temperament: high activity and briskness with considerable stimulation, high endurance, and low emotional reactivity; (3) phlegmatic temperament: low activity and briskness, high endurance, and low emotional reactivity; (4) melancholic temperament: low activity and briskness with limited stimulation, low endurance, and high emotional reactivity.

### Attributional Theory

In recent years, numerous theorists have proposed applying social psychology to explore human behavior based on the premise that social psychology can play a key role in personality and social environments. For example, the internal motivation and external motivation of individuals can be studied as resulting from their social environment, and then their influence can be used to explain specific differences between people (Amabile, 1988, 1990). Moreover, a social environment can affect individual differences, which can be explained as individual differences in a persistent motivation orientation (Deci and Ryan, 1985).

On the basis of self-perception of internal and external motivations, Bern (1967, 1972) argued that individuals must explain their attitude and motivation through their own behavior and its occurrence. For the relationship between teaching evaluation of teachers and learning motivation of students, Weiner’s (1986) attribution theory can explain the causal trajectory (internal and external factors), stability and controllability. The internal and external attribution theory was subsequently applied in cross-cultural teaching research. For example, by comparison of teaching models in Germany and China, researchers discovered that evaluation values of teachers in classroom settings in both countries directly affect the emotions and learning motivations of their students; specifically, the examined Chinese students were influenced by the achievement effect, whereas the examined German students were not (Zhou and Urhahne, 2013). The aforementioned study highlighted the influence of cultural differences. With regard to factors that affect the learning motivation of students, Chinese scholars have mainly explored this topic from the perspective of external and internal attribution (Chen et al., 2018). Given that learning motivation scales lack cross-cultural consistency, Chinese scholars subsequently developed a learning motivation questionnaire that was adapted to their cultural background. Among the scales that were developed, the most representative are those developed by Yumin et al. (1996); Huang and Zheng (1999), and Liu and Carless (2006), who developed motivation scales for assessing Chinese students in primary school, middle school, and university settings, respectively. Huang and Zheng’s (1999) college students’ learning motivation questionnaire was also based on the internal and external attribution theory. Chen (2018) subsequently used Huang’s questionnaire to study problems related to the self-perceived locus of control and learning motivation of college students.

## Materials and Methods

### Participants

The participants (*N* = 750) were college students from Sanming University; 47.5 and 52.5% of them were males and females, respectively, and 26 invalid and 724 valid questionnaires were collected. The participants were aged between 17 and 24 years (mean: 20 years); 28.5 and 46% of the participants were the only and the eldest child in their family, respectively. Their main fields of study were liberal arts, science, and other specialties (art, sports, and music). In addition, for this study, the participants were required to participate in a compulsory psychology course held by their college; the teaching framework of this course has remained consistent, but its teaching mode has changed because of China’s epidemic prevention and control measures in the 3 years following the start of the COVID-19 pandemic.

### Procedure

The questionnaires were administered and collected thrice. It was first distributed in the classroom between September and November 2019. During this period, an offline teaching mode was implemented in the classroom. Thereafter, it was administered online between March and May 2020. Because of the COVID-19 epidemic, the Ministry of Education of the People’s Republic of China (2020) released the Guidelines on Organization and Management of Online Teaching of General Institutions of General Institutions of Higher Education During Epidemic, which required schools to suspend offline classes and conduct online teaching to maintain the progress of classroom curricula. Therefore, the teaching mode during this period was limited to online teaching. The questionnaire was distributed in class for the third time between March and May 2021. During this period, teachers mainly followed the policy of implementing mixed teaching, specifically online–offline mixed teaching. For all the three surveys, the students voluntarily participated after understanding the research content of this study.

### Measures

#### Formal Personal Characteristics: Temperament Inventory

In China, temperament research is mainly based on Hippocratic temperament types and Pavlov’s theory of advanced neural activity types (Liu and Chen, 1992). Zhang and Chen (1985) subsequently integrated the two aforementioned types with Chinese culture to develop a temperament scale that is suitable for use in China. The scale uses the four temperaments as classified by Hippocrates. In addition, on the basis of Pavlov’s four basic types of neural activity (i.e., strong imbalance, strong balance and flexibility, strong balance inflexibility, and weak type), a new scale was developed. In the scale, each temperament type was covered by 15 questions, and the scale was tested and revised twice in a follow-up. The reliability of the second test was more than 0.6 (Lu et al., 1994). Subsequently, the scale was retested again, and the item discrimination of each topic was tested to verify the reliability and validity of the scale (Lu and Wang, 1995). In the course on psychology, the scale was also part of the course curriculum. Therefore, during the implementation of this study, 65.7% of the participants were determined to have one temperament type, whereas the remaining 34.3% were determined to have two or more temperament types. Among the participants, 20% had a choleric temperament, 51.5% had a sanguine temperament, 49.6% had a phlegmatic temperament, and 21.7% had a melancholic temperament.

#### Formal Behavioral Characteristics: Learning Motivation Questionnaire for College Students

This study adopted the learning motivation questionnaire for college students that was developed by Huang and Zheng (1999). Widely used in China, this questionnaire was designed to be used mainly in a Chinese cultural environment, and it considers the relationship between individuals and their external environments. It assumes that college students have six types of needs in learning, namely, physiology, safety, communication, respect, development, and contribution. The 26-question questionnaire assesses the intensity of six learning motives of college students (i.e., knowledge seeking and enterprise, social orientation, material pursuit, fear of failure, personal achievement, and small group orientation; Chen et al., 2018). For the original questionnaire, a four-level evaluation standard was applied. For the first time, 1,035 college students from various universities were examined by performing an analysis of variance (1999). Thereafter, Chen et al. (2018) tested the item reliability and validity of 602 questionnaires using a six-level evaluation standard. The internal consistency reliability coefficient of their scale was 0.89: the higher the total score was for learning motivation, the stronger the motivation intensity of a participant was. This study used a later version of the questionnaire. The reliability of the original data was favorable (α = 0.892).

## Results

### Comparison of Learning Motivation of Four Temperament Types Under Various Teaching Modes

This study used the SPSS statistics software to measure the collected data. The questionnaire used to measure different people’s learning motivations had a reliability (α) value of 0.893, which was greater than 0.7 (the accepted standard threshold; Hair et al., 2020; Su and Wu, 2021), thereby verifying the reliability of the questionnaire.

The ANOVA results revealed that the learning motivation of the students was influenced by their personal temperament and the teaching modes that they accepted. In terms of learning motivation, people with melancholic and choleric temperaments tended to have a high *F*-value, particularly those with a melancholic temperament. Furthermore, people with choleric and sanguine temperaments and those with choleric and phlegmatic temperaments had *F*-values that were also significant. The *F*-values of those who had both phlegmatic and sanguine temperaments, choleric and phlegmatic temperaments, and choleric temperament and sanguine temperaments also had highly significant results when specific teaching methods were applied (Table 1B).

**TABLE 1B:** Comparison of learning motivations by temperament type and teaching mode (analysis of variance).

Ind. variable	SS	*df*	MS	*F*	*p*
CLR	984.837	1	984.837	4.115*	0.043
SG	168.397	1	168.397	0.704	0.402
PHG	414.246	1	414.246	1.731	0.189
MLC	3,915.398	1	3,915.398	16.360***	0.000
TM	943.741	2	471.871	1.972	0.140
CLR * SG	1,676.657	1	1,676.657	7.005**	0.008
CLR * PHG	1,227.404	1	1,227.404	5.128*	0.024
CLR * MLC	41.008	1	41.008	0.171	0.679
CLR * TM	467.423	2	233.712	0.977	0.377
SG * PHG	1,744.648	1	1,744.648	7.290**	0.007
SG * MLC	448.286	1	448.286	1.873	0.172
SG * TM	230.554	2	115.277	0.482	0.618
PHG * MLC	589.515	1	589.515	2.463	0.117
PHG * TM	1,256.464	2	628.232	2.625	0.073
MLC * TM	487.609	2	243.804	1.019	0.362
CLR * SG * MLC	5.774	1	5.774	0.024	0.877
CLR * SG * TM	1,618.057	2	809.029	3.380*	0.035
CLR * PHG * TM	2,004.993	2	1,002.496	4.189*	0.016
CLR * MLC * TM	625.450	1	625.450	2.613	0.106
SG * PHG * TM	3,387.090	2	1,693.545	7.076**	0.001
SG * MLC * TM	11.850	1	11.850	0.050	0.824
PHG * MLC * TM	930.007	1	930.007	3.886*	0.049
CLR * SG * MLC * TM	100.051	1	100.051	0.418	0.518

CLR, choleric; SG, sanguine, PHG, phlegmatic, MLC, melancholic; TM, teaching mode. *p < 0.05, **p < 0.01, and ***p < 0.001.

Learning motivation was mainly classified into six dimensions (Huang and Zheng, 1999). This study used the six dimensions as dependent variables to evaluate their effect under the influence of multiple factors. Because of length constraints, this study only lists significant differences in independent variables (Table 2). The results revealed that the six dimensions (i.e., knowledge seeking and enterprise, social orientation, material pursuit, fear of failure, personal achievement, and small group orientation) were affected by multiple factors, meaning that different temperament types exhibited differences under different teaching modes. The various teaching methods resulted in significant differences in the three dimensions of knowledge seeking and enterprise (15.583, *p* < 0.001), personal achievement (8.164, *p* < 0.001), and small group orientation (8.069, *p* < 0.001). The F values of knowledge seeking and enterprise, social orientation, material pursuit, and personal achievement were significant for various types of personal temperament, especially for social enterprise (9.218, *p* < 0.01), material pursuit (8.147, *p* < 0.01), and personal achievement (12.915, *p* < 0.001); significant differences were detected between individuals with and without a melancholic temperament. The students with a phlegmatic temperament or melancholic temperament who had low activity levels exhibited differences in their ability to cope with personal knowledge seeking and personal achievement (3.935 for phlegmatic temperament, *p* < 0.05; 5.902 for melancholic temperament, *p* < 0.05), but this difference only applied to material and external pursuits. The students with sanguine and phlegmatic temperaments who had similar levels of reaction speed, patience, and emotional response exhibited differences under different teaching modes (4.359 for sanguine temperament, *p* < 0.05; 4.913 for phlegmatic temperament, *p* < 0.01). For fear of failure and personal achievement, the students with choleric and sanguine temperaments had high reaction speeds, which were also different under different teaching modes (5.651 for choleric temperament, *p* < 0.01; 3.797 for sanguine temperament, *p* < 0.05).

**TABLE 2:** Comparison of learning motivations by dimension, temperament type, and teaching modes (analysis of variance).

Dep. variable	Source	SS	*df*	MS	*F*	*p*
KSE	CLR	86.962	1	86.962	5.654*	0.018
	PHG	111.657	1	111.657	7.259**	0.007
	MLC	321.400	1	321.400	20.895***	0.000
	TM	479.374	2	239.687	15.583***	0.000
	SG*PHG	103.587	1	103.587	6.735*	0.010
	SG*MLC	67.380	1	67.380	4.381*	0.037
	PHG*MLC	126.406	1	126.406	8.218**	0.004
	PHG*MLC*TM	60.520	1	60.520	3.935*	0.048
SO	MLC	236.270	1	236.270	9.218**	0.002
	CLR*SG*TM	198.033	2	99.017	3.863*	0.021
	SG*PHG*TM	223.486	2	111.743	4.359*	0.013
MP	CLR	77.667	1	77.667	6.178*	0.013
	MLC	102.415	1	102.415	8.147**	0.004
	CLR*PHG	54.517	1	54.517	4.337*	0.038
	SG*PHG	114.985	1	114.985	9.147**	0.003
	PHG*MLC	53.367	1	53.367	4.245*	0.040
	SG*PHG*TM	123.513	2	61.756	4.913**	0.008
FOF	CLR*SG	95.635	1	95.635	5.115*	0.024
	CLR*PHG	100.882	1	100.882	5.396*	0.020
	CLR*PHG*TM	211.312	2	105.656	5.651**	0.004
	SG*PHG*TM	172.528	2	86.264	4.614*	0.010
PA	MLC	111.913	1	111.913	12.915***	0.000
	TM	141.484	2	70.742	8.164***	0.000
	CLR*SG	90.914	1	90.914	10.492**	0.001
	SG*PHG	54.507	1	54.507	6.290*	0.012
	CLR*PHG*TM	65.802	2	32.901	3.797*	0.023
	MLC*PHG*TM	51.139	1	51.139	5.902*	0.015
SGO	TM	121.736	2	60.868	8.069***	0.000
	PHG*TM	67.804	2	33.902	4.494*	0.012

LM, learning motivation; KSE, knowledge seeking and enterprise; FOF, fear of failure; SO, social orientation; MP, material pursuit; PA, personal achievement; SGO, small group orientation. *p < 0.05, **p < 0.01, and ***p < 0.001.

### Differences in Learning Motivation Under Different Teaching Modes

In this study, three teaching modes were examined, namely, traditional classroom teaching (*N* = 246), online teaching (*N* = 231), and online–offline mixed teaching (*N* = 247). The results obtained by multiple comparisons using the least significant difference method revealed differences between traditional classroom teaching and online teaching for learning motivation. Compared with traditional teaching, online teaching resulted in increased learning motivation among students. However, no difference was detected between mixed teaching and the other teaching modes (i.e., online teaching and traditional classroom teaching; Table 3).

**TABLE 3:** Comparison of learning motivation under various teaching modes.

(I)GROUP *(J)GROUP	MD(I-J)	SE	*p*	Ind. variable	Mean	*SD*	SEM
TCT*OT	2.8735*	1.41739	0.0906	TCL	113.7480	16.74714	1.06776
TCT*MT	1.0961	1.39351	–1.6399	OT	110.8745	16.42524	1.08070
OT*MT	–1.7774	1.41600	0.210	MT	112.6518	15.63637	0.99492

TCT, traditional classroom teaching; OT, online teaching; MT, mixed teaching. *p < 0.05.

Overall, students of all temperaments (more than half of all the examined students) liked mixed teaching (Table 4). The students preferred traditional classroom teaching to online teaching; however, among the students with a melancholic temperament, this difference was not large. Specifically, the proportion of students who liked mixed teaching was lower in the melancholic temperament group than in the other temperament groups.

**TABLE 4:** Comparison of teaching modes preferred by students of various temperament types.

Temperament	*N*	TCT (%)	OT (%)	MT (%)
CLR	149	27.52	7.38	65.10
SG	373	20.91	10.46	68.63
PHG	358	17.32	13.97	68.99
MLC	157	19.75	17.83	62.42

### Influence of Individual Temperament and Gender on Learning Motivation

During the *t*-test process (Table 5), differences in temperament type and gender led to varying levels of differences in learning motivation. Overall, significant differences in learning motivation, knowledge seeking and enterprise, and fear of failure were detected between students with and without a choleric temperament. In terms of average values, the students without a choleric temperament paid more attention to the three aforementioned aspects, meaning that the students with a choleric temperament were less afraid of failure (*t* = 2.243, *p* < 0.05). The students with a sanguine temperament focused more on social orientation (*t* = –2.072, *p* < 0.05) and personal achievement (*t* = –2.296, *p* < 0.05) than the students without a sanguine temperament. The students with a phlegmatic temperament focused more on learning and enterprise than those without a phlegmatic temperament (*t* = –3.547, *p* < 0.001); the *t* value of the students without a phlegmatic temperament were higher than that of the students with a phlegmatic temperament for knowledge seeking and enterprise (*t* = 3.697, *p* < 0.001). The students with a melancholic temperament only differed from the students with other temperaments in terms of fear of failure and small group orientation; however, they paid little attention to learning, knowledge-seeking, social orientation material pursuit, and personal achievement. Compared with the male students, the female students paid more attention to knowledge, enterprise (*t* = –2.312, *p* < 0.05), and social orientation (*t* = –2.499, *p* < 0.05), whereas the male students paid more attention to personal achievement (*t* = 3.492, *p* < 0.01).

**TABLE 5:** Comparison of learning motivation by temperament and gender (*t*-test).

Source	Verified variable	Attribute	*N*	*M*	*SD*	*t*
CLR	LM	N	575	113.3113	15.86228	2.784**
		Y	149	109.1611	17.52337	
	KSE	N	574	28.70	4.200	2.173*
		Y	149	27.85	4.647	
	FOF	N	575	13.78	4.530	2.243*
		Y	149	12.87	4.103	
SG	SO	N	346	25.40	5.171	–2.072*
		Y	373	26.21	5.235	
	PA	N	351	12.07	3.233	–2.296*
		Y	373	12.61	3.097	
PHG	KSE	N	365	27.97	4.669	–3.547***
		Y	358	29.09	3.826	
	PA	N	365	12.78	3.199	3.697***
		Y	359	11.91	3.090	
MLC	LM	N	567	113.5238	16.20482	3.372**
		Y	157	108.6051	16.07385	
	KSE	N	566	28.83	4.142	3.353**
		Y	157	27.44	4.710	
	SO	N	567	26.28	5.280	5.030***
		Y	152	24.10	4.596	
	MP	N	567	19.64	3.644	2.418*
		Y	157	18.83	4.003	
	PA	N	567	12.51	3.140	2.667**
		Y	157	11.75	3.230	
Gender	KSE	Male	344	28.14	4.317	–0.2.312*
		Female	380	28.88	4.273	
	SO	Male	344	25.31	5.276	–2.499*
		Female	380	26.28	5.124	
	PA	Male	344	12.78	3.103	3.492*
		Female	380	11.96	3.190	

LM, learning motivation; KSE, knowledge seeking and enterprise; FOF, fear of failure; SO, social orientation; MP, material pursuit; PA, personal achievement; SGO, small group orientation. *p < 0.05, **p < 0.01, and ***p < 0.001.

### Structural Model

**FIGURE 1:**
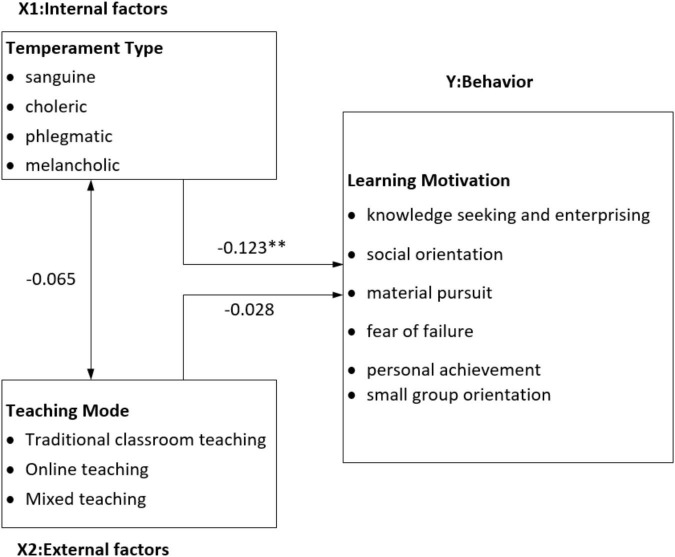
Research model.

Figure 1 reveals that the effects of interactions between temperament types and the external environment on behavior resulted in significant differences in learning motivation among the students with different temperaments; furthermore, a negative correlation between internal factors and behavior was also revealed (–0.123). Among the students with various temperaments (from choleric to melancholic), those with a melancholic temperament paid more attention to learning motivation. Although this study revealed that teaching mode was not associated with significant differences in temperament type and learning motivation, it also discovered a negative correlation trend. This may be caused by the weaker learning motivation of students in a traditional classroom setting and the more pronounced effects of traditional teaching on students with a choleric temperament.

## Discussion and Conclusion

This study evaluated the learning motivation of students with different types of temperaments who were exposed to different teaching environments and different teaching modes that were implemented over three time periods spanning the time before and after the epidemic (i.e., traditional classroom teaching period, online teaching period, and offline–online mixed teaching period). Existing theories assume the presence of a ternary interaction between the environment and the individual and their behavior (Bandura, 1986). However, in this study, a clear ternary interaction was not observed, indicating that the students with a melancholic temperament and low activity levels had weak learning motivations. This result partially supports the existing research hypothesis that personal temperament affects the learning motivation of students (Rawlings et al., 2017), and the weak learning motivation of students with a melancholic temperament was mainly reflected in their pursuit of external knowledge, social orientation, material pursuit, and personal achievement, which are associated with their low activity level, slow response, and low tolerance level (Bojanowska and Zalewska, 2016). The aforementioned elements must be removed by students from their external environment. Although the students with a melancholic temperament had highly emotional responses and were sensitive to changes in external information, they were prone to giving up when they encountered difficulties in receiving such information because of their low patience and slow response. This finding indicates that temperament type, as a personal factor, can affect the behavioral motivation of an individual.

Because of their slow response and low activity, the enthusiasm of the students with a melancholic temperament was slightly lower than that of the students without a melancholic temperament when they were under the diverse mixed teaching mode. As long as their teachers used the traditional didactic teaching method for either offline or online teaching, the learning and learning motivation of the students with a melancholic temperament were not affected. This was because the didactic teaching mode is highly consistent with the passive acceptance mode that is preferred by students who are influenced by Confucianism; these students do not require high levels of interaction (Fu et al., 2022), and the didactic method remains unchanged regardless of whether the online or offline teaching mode is implemented. The teachers’ teaching method essentially remained unchanged, and it was still implemented on the basis of the original preference for indoctrination. Although this teaching method appeared to switch from the traditional offline mode to the online mode, its focus on improving the memory of students remained unchanged (Sakurai et al., 2014). Although students with a melancholic temperament can be sensitive to changes in their teaching environment, online teaching is similar to traditional teaching in that it does not require students to participate in numerous activities or to improve their ability to adapt to changes. Therefore, the students with a melancholic temperament did not exhibit a particular preference for either of these two teaching modes. In contrast, in several Western countries, the COVID-19 pandemic forced teachers to implement online teaching, which is a teaching style that differs substantially from their original teaching styles. In Western countries such as South Africa, Wales, and Hungary, teachers could originally choose from numerous teaching practices, but with online teaching becoming the only option, differences emerged in terms of the learning participation of students (Cranfield et al., 2021).

Eastern countries are influenced by Confucian culture. Temperament is based on natural physiological factors, and it is difficult to change in an acquired environment. Through three random experiments, this study revealed that most of the students had a sanguine or phlegmatic temperament. People with a sanguine or a phlegmatic temperament are similar in that they respond quickly, exhibit high tolerance levels, and have low emotional responses (Bojanowska and Zalewska, 2016), meaning that they experience limited emotional changes and focus on how information is processed by their minds. Every culture has its unique aspects. Confucianism advocates the core value of benevolence and the cultural characteristic of collectivism (Chin, 2014); thus, teachers with these beliefs undertake the responsibility of maintaining the relationship between their surrounding environment and individuals, and they are accustomed to implementing the relevant standards and principles in a social and political community; this is generally applicable in the field of humanities and social sciences (Chin et al., 2021b). Therefore, Chinese teachers care greatly about their students. This form of care can be easily implemented through an education mode in which the concept of “thinking for the students” is emphasized in an offline classroom; in this way, students become accustomed to this passive, unitary, and fixed educational concept. Furthermore, teachers are concerned about how they can respond in a timely manner and maintain long-term concentration in this mode. These characteristics play a role in memory improvement. For memory, the stability of attention switching between emotional and non-emotional has a certain influence on memory (Koster et al., 2013). Teachers with a serious and responsible attitude attach great importance to the feedback of students regarding the memorization process of learning. In the long term, students adapt themselves to meet the needs of the collective because of the requirements of their external environment. Thus, controlling and stabilizing emotions has become a key factor that influences their personalities. When children grow up in an extended postnatal environment, they tend to develop the two temperaments that are primarily characterized by emotional stability (i.e., the phlegmatic and sanguine temperaments).

Therefore, in a collective culture that emphasizes didactic teaching, students who are active and cheerful (i.e., sanguine temperament) and those who are quiet and calm (phlegmatic temperament) tend to prefer the traditional teaching mode, especially those with a sanguine temperament. Because of their strong personality and high level of patience, students who were learning in offline classrooms had a stronger sense of collective activity than those who were learning in online classrooms. Moreover, this study revealed that, for learning motivation, the students with a sanguine temperament focused more on personal achievement and social orientation than those without a sanguine temperament, verifying that Chinese students focus on whether their values are reflected in their social groups (Liu et al., 2015). Therefore, under the influence of Confucianism, the students with a sanguine temperament tended to prefer the traditional teaching mode. Because of the influence of the traditional teaching mode, most randomly selected participants had a sanguine or phlegmatic temperament. The students with a choleric temperament also had high activity levels and fast responses, but they were less patient than those with a sanguine or phlegmatic temperament; furthermore, their emotional response was slower, they did not perform well in terms of emotion regulation, and they tended to experience mood fluctuations due to changes in their external environment. Therefore, in addition to mixed teaching, most of these students tended to prefer traditional offline teaching. Their long-term exposure to traditional classroom teaching also made them emotionally adaptable. Therefore, compared with the students without a choleric temperament (including those with a melancholic temperament), those with a choleric temperament preferred the traditional teaching style, and only 7.38% of them preferred online teaching. In summary, a two-way relationship exists between the students’ personal temperament factors and their external teaching environment.

Among the three teaching modes that were examined in this study, the strongest learning motivation among the students was achieved through traditional offline teaching. Although this was an unexpected finding, it is consistent with those of other studies, which have found that Chinese students who are influenced by Confucian culture prefer traditional teaching (Chan, 2019). In contrast, students in Western countries prefer diversified and mixed teaching, followed by online teaching. These findings indicate that the teaching mode of an external environment affects an individual’s learning motivation. However, in this study, this effect was not pronounced, especially for mixed teaching, which barely changed the learning motivation of the students; this was because mixed teaching integrates various teaching styles. However, compared with the other two modes, online teaching was associated with the lowest level of learning motivation. This finding can serve as a reference for efforts to increase the willingness of teachers to participate in online teaching after the epidemic. For example, given the finding of this study that students with a sanguine temperament value social orientation, we can consider how the social support aspect of online teaching can be enhanced. The aforementioned phenomenon occurs because during the learning process, individuals interact and create by organizing information in their brain and from their external environment. However, when an individual discovers that people around them are using the same resources, interactions tend to occur in an unpleasant and tense climate (Chin et al., 2020a,b). Methods for improving the learning habits, adaptation skills, and identification abilities of students should be explored because students with a choleric or melancholic temperament are highly sensitive and exhibit low levels of patience. When these students encounter numerous obstacles in online learning, they tend to give up because of their inability to adapt. Finally, because students with a phlegmatic temperament emphasize knowledge seeking, how to simplify online learning and enhance its usefulness is also a topic that should be further explored. Our findings have indicated factors that teachers must focus on if they want to continue to conduct online teaching after the epidemic (Mo et al., 2021). Although online teaching was the favorable teaching mode during the epidemic period, this study revealed that students were still more accustomed to the traditional teaching method.

The learning motivation of students conflicts the teaching mode promoted by the current social environment, especially the online teaching mode that has emerged because of the current epidemic, and that is being promoted by Western countries. When cultural differences between Eastern and Western thinking arise (e.g., Chinese traditional teaching vs. Western diversified online teaching), a problem of individual cognition emerges. This is because Confucianism integrates the concept of Yin–Yang harmony that is emphasized in *I Ching*; Yin represents passive energy, whereas Yang represents positive energy (Chin et al., 2020a,b). When an attribute of Yin or Yang attains a certain level of energy, the opposing attribute is also born (Lloyd, 1996). Thus, when the traditional receptive teaching mode reaches a certain level of development, some students will begin to transition gradually and pursue unrestrained and free active learning. The online teaching mode can meet this demand. If teachers want to continue to recommend that students use online platforms for learning, they must improve the quality of their course content, because such content can encourage students to learn and enhance their satisfaction with their learning (Jin et al., 2021; Wang et al., 2021); this strategy is consistent with the finding that students with a sanguine temperament focus on knowledge seeking, and that students with a phlegmatic temperament are motivated by self-achievement.

In summary, an individual’s personal temperament is related to their external cultural atmosphere. Although temperament is primarily influenced by congenital factors, long-term exposure to Confucian culture, in which a collective didactic receptive teaching model is implemented, causes an individual to develop the adaptation mechanism of emotional stability. This temporal cultural background also indirectly affects the personality temperament of an individual. Confucian culture is conducive to the development of phlegmatic and sanguine temperaments. Individuals with choleric and melancholic temperaments are prone to emotional sensitivity and fluctuations and are limited in their ability to deal with changes in their external environment consequently, they are accepting of various teaching modes, and their learning motivation presents different forms of changes. However, because of the current epidemic and the influence of foreign diversified teaching models, students are generally adapted to traditional teaching but also tend to choose diversified modes in response to the requirements of a new environment, which also indirectly affects the learning motivation of students with different types of temperaments.

### Limitations and Future Directions

This study has several limitations. The theoretical basis of the study is TRD; however, this theory can only explain unitary interactions, which refer to the interrelation between temperament and environment. Although this study did not reveal an obvious direct correlation, it provided clear evidence that the Confucian environment of China causes most Chinese students to develop a sanguine or phlegmatic temperament, and that these temperament types are responsive to traditional teaching. However, this study did not explore the cultural differences between China and Western countries in the context of various temperament types. In addition, for the other two elements of interaction, a one-way relationship was found. This study verified the effects of personal factors on the learning motivation of students, but it did not verify how learning motivation affects personal factors. Teaching mode influenced the learning motivation of students but not the other way around, and even this direct relationship was weak. We could only use temperament type to evaluate how the learning motivation of students changes when a given teaching mode is implemented. Ultimately, a teaching mode plays an intermediary role rather than a direct role. Therefore, in our future research, we can explore how the temperament type of students can be evaluated on the basis of their motivation preferences; suitable learning styles can be selected for them according to their temperament type; thus, the focus can be expanded from the teaching environment in a given culture. This research direction can provide meaningful results and further the objective of implementing student-oriented education.

## Data Availability Statement

The raw data supporting the conclusions of this article will be made available by the authors, without undue reservation.

## Author Contributions

All authors contributed to the conception of the idea, implementation and analysis of the experimental results, and writing of the manuscript, and read and approved the final version of the manuscript.

## Conflict of Interest

The authors declare that the research was conducted in the absence of any commercial or financial relationships that could be construed as a potential conflict of interest.

## Publisher’s Note

All claims expressed in this article are solely those of the authors and do not necessarily represent those of their affiliated organizations, or those of the publisher, the editors and the reviewers. Any product that may be evaluated in this article, or claim that may be made by its manufacturer, is not guaranteed or endorsed by the publisher.
